# Supplementary material for: Saporin Toxin Delivered by Engineered Colloidal Nanoparticles Is Strongly Effective against Cancer Cells
Source: Pharmaceutics. 2022 Jul 21;14(7):1517. doi: 10.3390/pharmaceutics14071517 (PMC9319684; doi:10.3390/pharmaceutics14071517)
Supplement: Supplementary file 1 [file pharmaceutics-14-01517-s001.zip › pharmaceutics-1825690-supplementary.pdf]

Saporin toxin delivered by engineered colloidal nanoparticles is strongly effective against cancer cells

Lucia Salvioni †, Filippo Testa †, Linda Barbieri, Marco Giustra, Jessica Armida Bertolini, Giulia Tomaino, Paolo Tortora, Davide Prosperi and Miriam Colombo *

Department of Biotechnology and Bioscience, University of Milano-Bicocca, Piazza della Scienza 2, 20126 Milano, Italy
* Correspondence: miriam.colombo@unimib.it
† These authors contributed equally to this work.

Supporting information

Production and purification of C-Cys engineered-Saporin in E. coli

For this study, a recombinant version of SAP-3 isoform with the addition of a unique Cys residue located in the C-terminal of the protein [1,2] and an His-Tag in the N-terminus, was used. In particular, Saporin encoding plasmid was produced in house, amplifying Saporin gene by PCR and cloning it into SalI-NotI restriction sites of the pET30b plasmid. The reverse primer for the cloning also presented an addition of 3 nucleotides encoding for a Cys amino acid, upstream of the stop codon, to obtain an insertion of Cys residue. Primers: forward 5′-ATTA<u>GTCGAC</u>GTCACATCAATCACATTAGATCTAGT-3′, added SalI restriction site (underlined); reverse 5′-ATTA<u>GCGGCCGC</u>CTA**GCA**CTTTGGTTTGCCCAAATACATAAGG-3′, added NotI restriction site (underlined) and 3 nucleotides for Cys residue (bold).

Afterwards, E. coli strain BL21(DE3) was transformed using pET30b/Saporin plasmid and grown at 37 °C in Luria Bertani 50 μg/mL kanamycin medium. The strain was induced with 0.5 mM isopropyl β-D-1-tiogalactopiranoside at Abs 600nm 0.8. After 2.5 h, the cells were recovered by centrifugation (4000 xg, 15 min) and the pellet was resuspended in lysis buffer (5 mL/g wet weight; 50 mM potassium phosphate, pH 7.2, 150 mM NaCl, 1 mM phenylmethanesulfonyl fluoride, 1 mM DTT, 10 mM imidazole, 1 mg/mL lysozyme and 20 μg/mL DNAse I). The cell suspension was incubated in ice for 45 min and subsequently sonicated at 10% power three times, 30s pulse-on plus 30s pulse-off. The crude extract was centrifuged for 30 min at 27000 xg 4 °C and the supernatant loaded onto Ni Sepharose® 6 Fast Flow affinity column (1 mL bed volume, GE Healthcare), previously equilibrated with 10 volumes of buffer (50 mM potassium phosphate, pH 7.2, 150 mM NaCl, 10mM imidazole). The resin was incubated with the supernatant for 1 h at 4 °C, then fraction 1 was eluted (excluded raw extract), the resin was washed with 10 volumes of wash buffer (50 mM potassium phosphate, pH 7.2, 150 mM NaCl, 25 mM imidazole) and fraction 2 was eluted (washing 1). To elute Saporin, 4 fractions with increasing imidazole concentrations were eluted, respectively 100 mM, 300 mM, 500 mM and 1 M for fractions 3, 4, 5 and 6. Finally, the resin was washed with 10 volumes of 20 mM MES buffer pH 5 and fraction 7 was eluted (washing 2). The eluted protein was washed by Amicon® Ultra centrifugal filter (10 kDa) using 50 mM potassium phosphate, pH 7.2, 150 mM NaCl buffer to remove the excess of imidazole. Purified protein was collected, quantified by Micro BCA™ Protein Assay Kit (Thermo Fisher Scientific, Waltham, MA, USA) and stored at -20°C. The presence of Saporin and the purity of the different fractions were tested using 12% (v/v) polyacrylamide gels respectively by Western Blot analysis with His-Tag Monoclonal antibody (Proteintech Group Inc., Manchester, United Kingdom) and by sodium dodecyl sulfate - polyacrylamide gel electrophoresis (SDS-PAGE) with Imperial™ Protein Stain (Thermo Fisher Scientific, Waltham, MA, USA) staining.

MYTS-Sap binding specificity

The specificity of the MYTS-Saporin binding was evaluated by Western Blot. Briefly, MYTS-Sap-1, MYTS-Sap-2 and MYTS-Sap-3 were suspended in sample buffer (0.1 M Tris/HCl pH 6.8, 2% SDS, 20% glycerol) with or without the addition of the reducing agent β-mercapto-ethanol at similar concentrations of MYTS, corresponding to respectively 2 μg, 4 μg and 6 μg of protein/sample. Samples were separated by SDS-PAGE using 12% v/v polyacrylamide gels and then transferred onto polyvinylidene fluoride (PVDF) membrane. The membrane was blocked with 5% w/v non-fat milk in TBS (0.1% v/v Tween® 20) for 1 h. Then, the membrane was incubated overnight at 4 °C with mouse-monoclonal antibody against 6*-His-Tag (cat N. 66005-1-lg; Proteintech Group, Rosemont, IL, USA) at 1:15000 dilution in 5% w/v non-fat milk in TBS (0.1% v/v Tween® 20). The membrane was washed three times with TBS (0.1% v/v Tween® 20) and incubated for 1 h at RT with IRDye® 800CW Goat anti-Mouse IgG Secondary Antibody (1:15000 dilution; Li-Cor Inc, Lincoln, NE, USA) for 1 h. The fluorescence signal was detected at 800nm using the ChemiDoc™ System (Bio-Rad Laboratories Hercules, Inc, CA, USA).

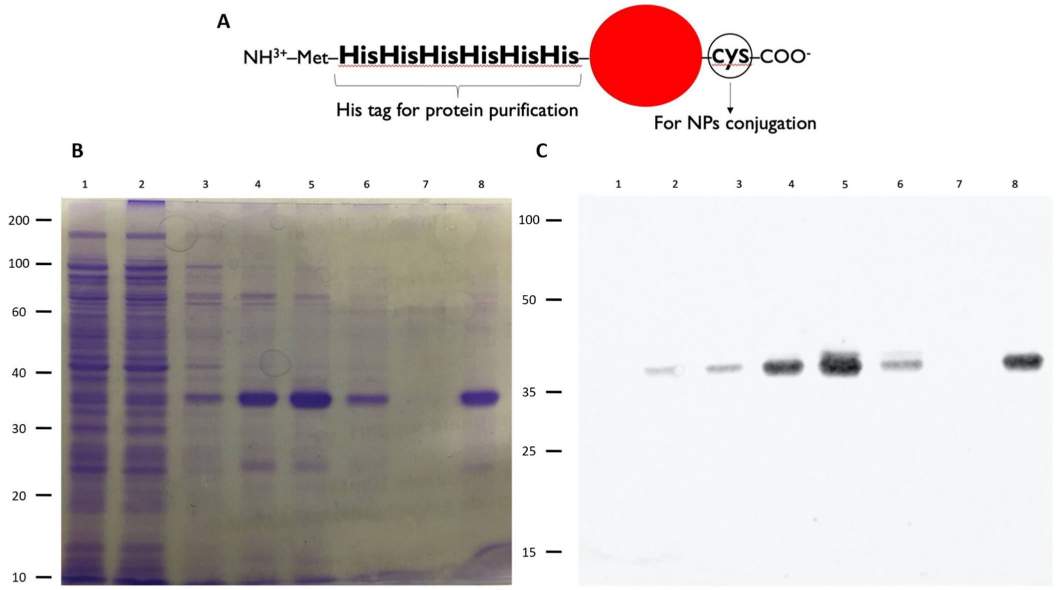

Figure S1. Production of recombinant Saporin. (A) Schematic representation of the recombinant protein used. (B) SDS-PAGE and (C) Western blot analysis (Primary Ab - Anti His-Tag Ab) of the fractions obtained from the protein purification done via affinity chromatography. 1: Excluded fraction; 2: Washing 1; 3: Imidazole 100 mM elution; 4: Imidazole 300 mM elution; 5: Imidazole 500 mM elution; 6: Imidazole 1 M elution; 7: Washing 2; 8: Positive control (Sap-His Tag).

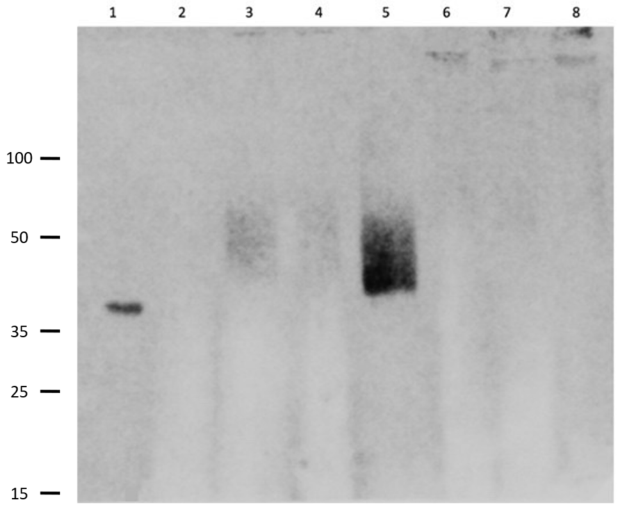

Figure S2. Assessment of MYTS-Sap binding specificity. The conjugation specificity of Saporin to MYTS was investigated with Western Blot analysis, both in reducing conditions (lane 3, 4 and 5 respectively for MYTS-Sap-1, MYTS-Sap-2 and MYTS-Sap-3 with addition of β-mercapto-ethanol) and in non-reducing conditions (lane 6, 7 and 8 respectively for MYTS-Sap-1, MYTS-Sap-2 and MYTS-Sap-3 without the addition of β-mercapto-ethanol). Lane 1: positive control; Lane 2: MYTS-SPDP.

Table S1. Correlation between Sap and MYTS concentrations in MTT assay. Saporin concentrations (nM) equivalent to MYTS concentrations (µg/mL) tested in the MTT assay on SK-BR-3 and NIH-3T3 cells.

	**C-Sap concentration (nM) corresponding to**		
	**25 µg/mL MYTS**	**37.5 µg/mL MYTS**	**50 µg/mL MYTS**
MYTS-Sap-1	13.5	20.2	26.9
MYTS-Sap-2	26.6	39.9	53.2
MYTS-Sap-3	55.5	83.3	111.1

References:

1. Fabbrini, M.S.; Rappociolo, E.; Carpani, D.; Solinas, M.; Valsasina, B.; Breme, U.; Cavallaro, U.; Nykjaer, A.; Rovida, E.; Legname, G.; et al. Characterization of a Saporin Isoform with Lower Ribosome-Inhibiting Activity. Biochemical Journal 1997, 322, 719–727, doi:10.1042/bj3220719.

2. Günhan, E.; Swe, M.; Palazoglu, M.; Voss, J.C.; Chalupa, L.M. Expression and Purification of Cysteine Introduced Recombinant Saporin. Protein Expression and Purification 2008, 58, 203–209, doi:10.1016/j.pep.2007.11.005.
